# Kappa Opioid Receptor Agonist Mesyl Sal B Attenuates Behavioral Sensitization to Cocaine with Fewer Aversive Side-Effects than Salvinorin A in Rodents

**DOI:** 10.3390/molecules23102602

**Published:** 2018-10-11

**Authors:** Bronwyn M. Kivell, Kelly F. Paton, Nitin Kumar, Aashish S. Morani, Aimee Culverhouse, Amy Shepherd, Susan A. Welsh, Andrew Biggerstaff, Rachel S. Crowley, Thomas E. Prisinzano

**Affiliations:** 1 School of Biological Science, Centre for Biodiscovery, Victoria University of Wellington, P.O. Box 600, Wellington, New Zealand; kelly.paton@vuw.ac.nz (K.F.P.); nitin23784@yahoo.com (N.K.); aashu110@gmail.com (A.S.M.); aimee.culverhouse@vuw.ac.nz (A.C.); shepheamy@gmail.com (A.S.); midgii@gmail.com (S.A.W.); Andy.Biggerstaff@vuw.ac.nz (A.B.); 2 Department of Medicinal Chemistry, University of Kansas, Lawrence, KS 6604, USA; crowleyrachels@outlook.com (R.S.C.); prisinza@ku.edu (T.E.P.)

**Keywords:** Salvinorin A, kappa opioid, locomotion, behavioral sensitization, conditioned taste aversion, forced swim test, depression, drug abuse, addiction, behavioral pharmacology

## Abstract

The acute activation of kappa opioid receptors (KOPr) produces antinociceptive and anti-cocaine effects, however, their side-effects have limited further clinical development. Mesyl Sal B is a potent and selective KOPr analogue of Salvinorin A (Sal A), a psychoactive natural product isolated from the plant *Salvia divinorum*. We assessed the antinociceptive, anti-cocaine, and side-effects of Mesyl Sal B. The anti-cocaine effects are evaluated in cocaine-induced hyperactivity and behavioral sensitization to cocaine in male Sprague Dawley rats. Mesyl Sal B was assessed for anhedonia (conditioned taste aversion), aversion (conditioned place aversion), pro-depressive effects (forced swim test), anxiety (elevated plus maze) and learning and memory deficits (novel object recognition). In male B6.SJL mice, the antinociceptive effects were evaluated in warm-water (50 °C) tail withdrawal and intraplantar formaldehyde (2%) assays and the sedative effects measured with the rotarod performance task. Mesyl Sal B (0.3 mg/kg) attenuated cocaine-induced hyperactivity and behavioral sensitization to cocaine without modulating sucrose self-administration and without producing aversion, sedation, anxiety, or learning and memory impairment in rats. However, increased immobility was observed in the forced swim test indicating pro-depressive effects. Mesyl Sal B was not as potent as Sal A at reducing pain in the antinociceptive assays. In conclusion, Mesyl Sal B possesses anti-cocaine effects, is longer acting in vivo and has fewer side-effects when compared to Sal A, however, the antinociceptive effects are limited.

## 1. Introduction

Drug addiction is a relapsing, compulsive disorder characterized by a lack of control in drug intake even when adverse consequences are apparent [1,2]. Psychostimulants, such as cocaine, are widely abused and currently, there are no Food and Drug Administration (FDA) approved treatments for psychostimulant addiction. Preclinical studies have demonstrated that kappa opioid receptor (KOPr) activation attenuates cocaine-induced behaviors such as cocaine self-administration [3,4], cocaine-enhanced locomotion [5,6], behavioral sensitization to cocaine [7,8], and the reinstatement of extinguished cocaine-seeking [9,10]. These effects of KOPr agonists can be attributed to their ability to decrease dopamine levels in the nucleus accumbens [11,12,13]. KOPrs have been shown to form oligomers with the dopamine transporter (DAT) and this interaction was also increased in the presence of Salvinorin A (Sal A) and results in an increase in the dopamine uptake by DAT [14]. KOPr activation in the ventral tegmental area also decreases dopamine levels in the medial prefrontal cortex [15]. These decreased dopamine levels within the reward pathways are likely responsible for reducing the rewarding effects of drugs of abuse. However, the side-effects of traditional KOPr agonists, such as depression [16], dysphoria [17,18], sedation [19], and aversion [20,21] have limited the therapeutic development of these agents. Several strategies have been employed to reduce the side-effects profile of KOPr agonists, including the development of G-protein biased agonists [22,23,24,25] and peripherally restricted KOPr agonists [26,27].

Sal A, derived from the hallucinogenic sage plant *Salvia divinorum* (Lamiaceae), is a potent and selective KOPr agonist [28,29]. Sal A has a novel neo-clerodane diterpene structure and shares similar pharmacological properties with the traditional KOPr agonists [30,31,32]. Previously, we have reported that Sal A and the analogues, 2-ethoxymethyl ether Sal B and β-tetrahydropyran Sal B, attenuate cocaine-prime induced cocaine seeking without inducing sedation or suppressing operant sucrose reinforcement in rats in a similar fashion to the traditional KOPr agonists U50488, U69593, and spiradoline [33,34]. However, Sal A has a rapid onset and short duration of the effects [35,36,37] attributed to the quick metabolism at the C-2 position to the metabolite Salvinorin B [29,35]. Sal A is also a substrate for the P-glycoprotein efflux pump [38]. Because of this poor pharmacokinetic profile, Sal A cannot be developed as an anti-cocaine agent. Structure-activity relationship studies involving Sal A suggests the C-2 position is important in the binding and activation of KOPr by Sal A [29,39,40] and is likely to be important in the design of analogues with improved pharmacokinetics [41,42]. Recently, C-2 substituted Sal A analogues have been synthesized with varying affinity for KOPr compared to Sal A [41,43]. Other pharmacological studies indicate that C-2 substituted Sal A analogues are longer acting [32,34,44]. Therefore, developing Sal A analogues with anti-cocaine properties, improved pharmacokinetics, and fewer side-effects are desirable for the development of potential anti-cocaine pharmacotherapies [32,45,46,47,48].

The Sal A analogue (2S,4aR,6aR,7R,9S,10aS,10bR)-9-(methanesulfonyloxy)-2-(3-furanyl)dodecahydro-6a, 10b-dimethyl-4,10-dioxy-2H-naptho [2,1-c]pyran-7-carboxylic acid methyl ester (Mesyl Sal B) has a mesylate substitution at the C-2 position and has been shown to have similar binding affinity for KOPr to Sal A and U69593 [41,45,49]. We have previously shown that Mesyl Sal B improved the pharmacokinetic effects in mice, using the warm-water tail withdrawal assay, and attenuated cocaine-prime induced cocaine seeking/taking in cocaine self-administering rats [24]. It has also been demonstrated that Mesyl Sal B attenuates the alcohol intake in mice following chronic escalation drinking [50].

Single and intermittent cocaine exposure produces an increase in the locomotion activity [51] and an increased locomotion sensitized response following a cocaine challenge in animals with prior cocaine experience [52,53,54]. The mesolimbic dopaminergic system has been implicated in cocaine-induced hyperactivity and behavioral sensitization and increased extracellular dopamine levels are observed within the ventral tegmental area and nucleus accumbens of rats receiving cocaine [55,56,57,58,59]. Since cocaine behavioral sensitization might play a part in craving and relapse to drug use [60,61,62], understanding the factors involved in attenuating behavioral sensitization to cocaine is likely to be useful for designing anti-cocaine therapeutics. Therefore, we tested the effect of Mesyl Sal B on cocaine-induced hyperactivity and the expression of cocaine behavioral sensitization in rats.

Furthermore, Mesyl Sal B was assessed for the side-effects associated with the traditional KOPr agonists, U50488 and U69593, as well as the parent compound Sal A. In laboratory animals, Sal A produces sedation and aversion [63,64,65], therefore, we tested the effect of Mesyl Sal B on rotarod performance in mice, and conditioned taste aversion (CTA) and conditioned place aversion (CPA) in rats. Since Sal A produces depressive effects [66], we also tested the effect of Mesyl Sal B on swimming behaviors using the forced swim test (FST), anxiogenic effects using the elevated plus maze (EPM), and cognitive performance using the novel object recognition task.

## 2. Results

### 2.1. Chemical Properties of Mesyl Sal B

**Figure 1:**
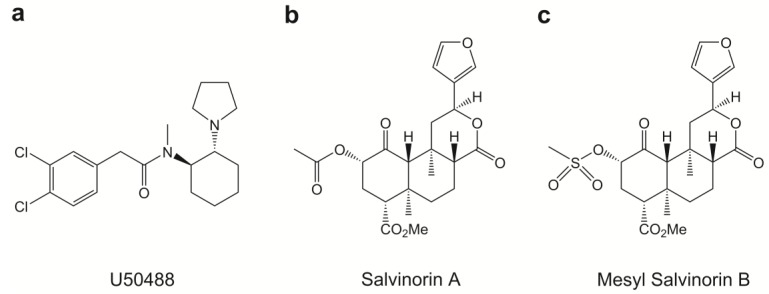
The chemical structures of (**a**) U50488, (**b**) Sal A and (**c**) Mesyl Sal B.

Mesyl Sal B is a novel Sal A analogue previously shown to bind KOPr with similar binding affinity (Mesyl Sal B, K_i_ = 2.3 ± 0.1 nM vs. Sal A, K_i_ = 1.9 ± 0.2 nM) in Chinese hamster ovary (CHO) cells stably expressing the human KOPr, using [^125^I]IOXY as the radioligand [41]; and similar potency (EC_50_ = 30 ± 5 nM) to Sal A (EC_50_ = 40 ± 10 nM) assessed using [^35^S]GTPγS functional assays in CHO cells [41,48] (Figure 1). Data from functional cellular assays measuring KOPr-mediated inhibition of forskolin-induced cyclic adenosine monophosphate (cAMP) accumulation (DiscoveRx HitHunter assays) and in assays evaluating β-arrestin2 recruitment are shown in Table 1. Assays evaluating signaling bias compare the potency and efficacy of test ligands (Mesyl Sal B) against reference ligands (U50488) in both G-protein dependent and independent assays. Here we use the cAMP and β-arrestin2 recruitment assays to evaluate signaling bias and show that Mesyl Sal B is a full agonist in both assays, and furthermore, to show that Mesyl Sal B has G-protein signaling bias.

### 2.2. Anti-Cocaine Effects of Mesyl Sal B

**Figure 2:**
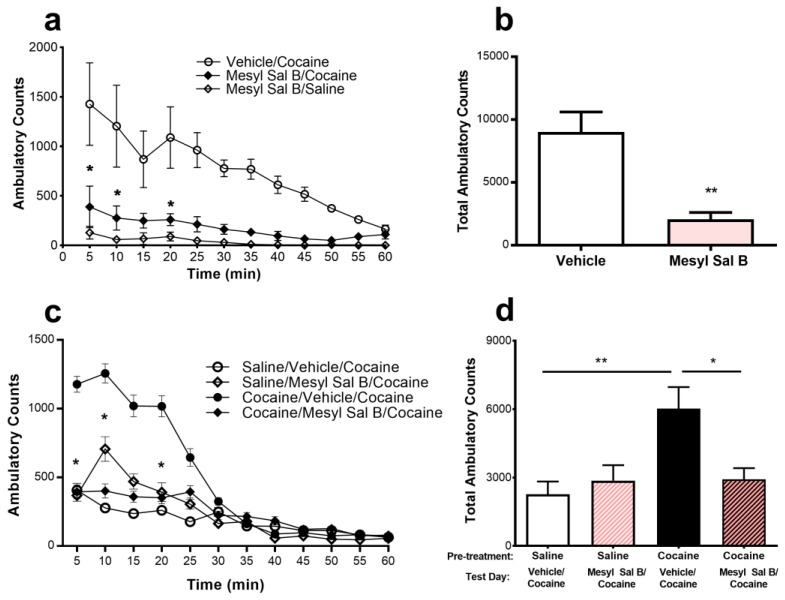
The effects of Mesyl Sal B on the expression of cocaine-induced hyperactivity and behavioral sensitization to cocaine were evaluated in rats. Pre-exposure to Mesyl Sal B (0.3 mg/kg, i.p.) 45 min prior to cocaine (20 mg/kg) significantly attenuated the cocaine-induced hyperactivity compared to vehicle-treated control rats: (**a**) Analysis of locomotor activity over time revealed significant effects of Mesyl Sal B administration on cocaine-induced hyperactivity at 5, 10, and 20 min. Two-way ANOVA followed by Bonferroni post hoc test. (**b**) Mesyl Sal B attenuated the total ambulatory counts observed in 60 min, Student *t*-test. (**c**) Rats were injected with saline (1 mL/kg) or cocaine (20 mg/kg) for 5 consecutive days then remained drug-free from days 6–9. On test day (day 10), rats were injected with either vehicle or Mesyl Sal B (0.3 mg/kg) and 45 min later they were injected with cocaine (20 mg/kg). Locomotor activity in 5 min intervals and (**d**) the total ambulatory counts over 60 min shows that Mesyl Sal B attenuates behavioral sensitization to cocaine. One-way ANOVA followed by a Bonferroni post hoc test. Data presented as mean ± standard error of the mean (SEM); n = 4–8 for each group (* *p* < 0.05; ** *p* < 0.01).

Studies to determine the effects of Mesyl Sal B on cocaine-induced hyperactivity in rats are shown in Figure 2. Mesyl Sal B (0.3 mg/kg) suppressed the cocaine-induced hyperactivity when administered 45 min prior to cocaine (Figure 2b). A time course of effects on suppression of cocaine-induced hyperactivity was performed between 5–60 min, with 45 min pre-incubation showing optimal effects (data not shown). The time-course analysis revealed a significant effect of Mesyl Sal B on cocaine-induced hyperactivity (F (3,168) = 23.64, *p* < 0.0001) at 5, 10, and 20 min (Bonferroni post hoc test; Figure 2a).

The effect of Mesyl Sal B (0.3 mg/kg) on the expression of cocaine sensitization in rats is shown in Figure 2c,d. Control cocaine-sensitized rats produced a significant increase in the locomotor activity on the test day (day 10) compared to the saline-treated rats indicating the expression of cocaine sensitization (F(3,23) = 6.15, *p* = 0.003). Cocaine–sensitized rats were given a single injection of Mesyl Sal B (0.3 mg/kg) on the test day had attenuated cocaine-induced hyperactivity. Two-way analysis of variance (ANOVA) with repeated measures of time indicated a significant effect of treatment (F(3,276) = 25.39, *p* < 0.0001) and treatment x time interaction (F(33,276) = 3.84, *p* < 0.0001). Further post hoc analysis showed a significant effect at 5, 10, 15, 20, and 25 min (*p* < 0.05) (Figure 2c). This data suggests that Mesyl Sal B suppresses the expression of cocaine behavioral sensitization in rats. No changes in stereotypic counts were observed between any of these groups (data not shown).

### 2.3. Thermal Antinociception

**Figure 3:**
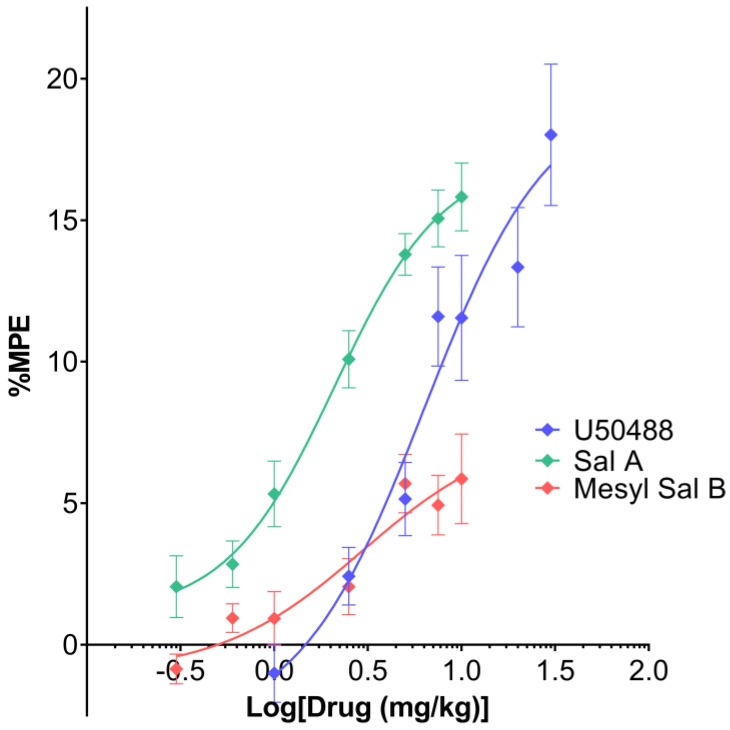
The effects of Mesyl Sal B on 50 °C warm-water tail withdrawal in mice showing cumulative dose-response effects. Non-linear regression analysis showed that a different curve fit each data set (F(6,93) = 14.43, *p* < 0.0001). Mesyl Sal B (EC_50_ = 3.0 mg/kg, E_max_ = 38% of U50488) exerts only partial antinociceptive effects in the tail withdrawal compared to both Sal A (EC_50_ = 2.1 mg/kg, E_max_ = 87% of U50488) and U50488 (EC_50_ = 6.7 mg/kg, E_max_ = 19.7 mg/kg). Data presented as mean ± SEM; n = 6 for each group.

Mesyl Sal B has previously been shown to have antinociceptive effects in the warm-water tail withdrawal assay in mice at 1 mg/kg, i.p. with a 60 min duration of action compared to 15 min for Sal A [24]. Results from in vitro cellular assays show that U50488, Sal A, and Mesyl Sal B are all full agonists at KOPr [41,48], therefore, in the present study, the warm-water tail withdrawal assay was used to assess the dose-response effects in vivo. A non-linear regression analysis showed that a different curve fit each treatment (F(6,93) = 14.43, *p* < 0.0001) (Figure 3). Mesyl Sal B (EC_50_ = 3.0 mg/kg) only exerted partial effects in the warm-water tail withdrawal assay in comparison to U50488 (EC_50_ = 6.7 mg/kg) and Sal A (EC_50_ = 2.1 mg/kg). Interestingly, the efficacy of Mesyl Sal B (E_max_ = 38%) was significantly reduced compared to the traditional agonist U50488 (*p* = 0.0064), whereas Sal A (E_max_ = 87%) was not significantly different to U50488 (*p* > 0.9999). Therefore, it appears that Mesyl Sal B has minimal effects on thermal antinociception, with a reduced potency and efficacy compared to both Sal A and U50488.

### 2.4. Effects of Mesyl Sal B on Intraplantar Formaldehyde Induced Inflammatory Pain

**Figure 4:**
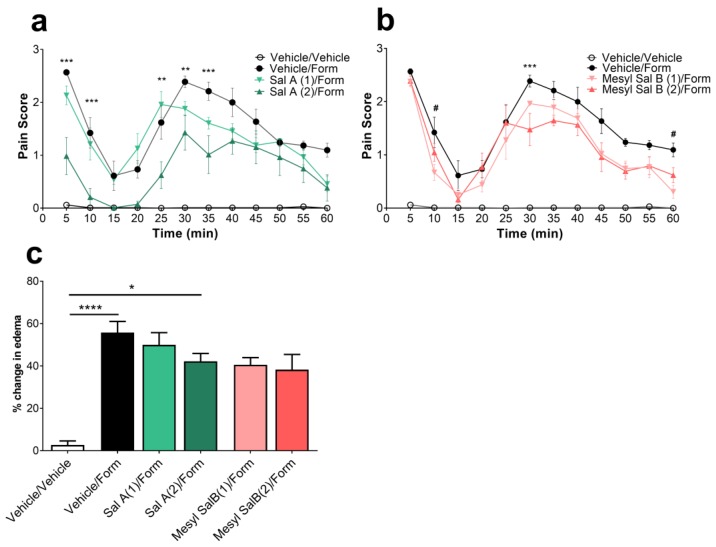
(**a**) The effects of Sal A and (**b**) Mesyl Sal B on phase 1 nociceptive pain (1–15 min) and phase 2 inflammatory pain (20–60 min). Sal A shows the significant attenuation of both phases of pain (F(33,220) = 3.169, *p* < 0.0001), whereas Mesyl Sal B exerts very limited attenuation of pain with significance seen at 10 min and 60 min for a 1 mg/kg dose, and at 30 min with a 2 mg/kg dose (F(33,220) = 4.28, *p* < 0.0001). (**c**) Paw swelling was evaluated 60 min following a 2% intraplantar formaldehyde with Sal A (2 mg/kg, i.p.) showing a reduction in the paw edema (F(5,60) = 16.72, *p* < 0.0001). Mesyl Sal B had no significant effect compared to the formaldehyde/vehicle-treated controls. Two-way repeated measures ANOVA followed by Bonferroni post hoc tests. Data presented as mean ± SEM; n = 6 for each group. Numbers in brackets indicate dose in mg/kg. (* *p* < 0.05; ** *p* < 0.01; *** *p* < 0.001, **** *p* < 0.0001 for the 2 mg/kg dose compared to the vehicle/formaldehyde treatment; # *p* < 0.05 for 1 mg/kg dose compared to vehicle/formaldehyde treatment). Form = formaldehyde.

To fully evaluate the effects of Mesyl Sal B on pain behaviors, we utilized the intraplantar 2% formaldehyde model of phase 1 nociceptive pain (0–15 min) and phase 2 inflammatory pain (20–60 min) in mice. While Sal A (2 mg/kg) showed significant attenuation of the phase 1 and phase 2 pain responses (F(33,220) = 3.169, *p* < 0.0001) (Figure 4a), Mesyl Sal B was minimally effective at attenuating phase 1 at 2 mg/kg dosages (F(33,220) = 4.28, *p* < 0.0001), reducing phase 2 pain at the 30 min time point for the 2 mg/kg dose (*p* = 0.0009; Figure 4b). The lower 1 mg/kg dose of Mesyl Sal B dose showed a small but significant decrease at 10 (*p* = 0.0104) and 60 min (*p* = 0.0059). The limited effectiveness of Mesyl Sal B in this nociceptive and inflammatory pain is consistent with no significant reduction in the formaldehyde-induced edema, whereas the 2 mg/kg dose of Sal A significantly reduced the paw edema (*p* = 0.0426; Figure 4c).

### 2.5. Side-Effect Profile of Mesyl Sal B

#### 2.5.1. Effects of Mesyl Sal B on Sucrose Self-Administration

**Figure 5:**
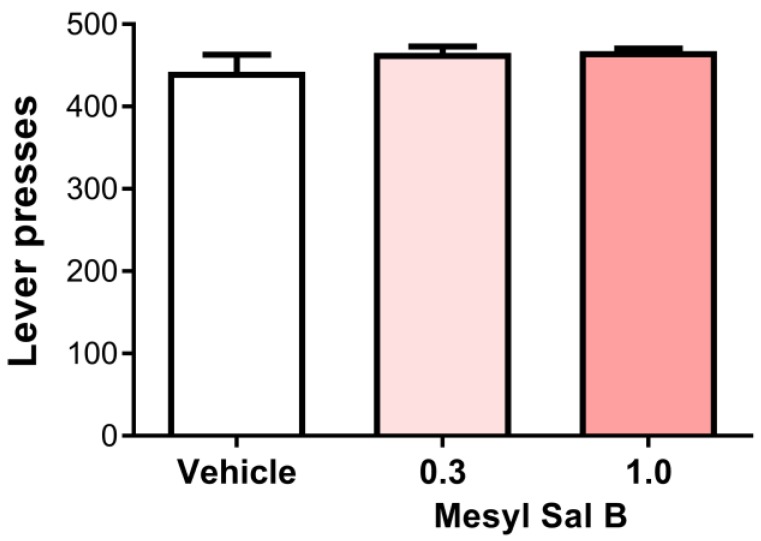
The effects of Mesyl Sal B on sucrose-reinforced responding. (**a**) Mesyl Sal B (0.3, 1.0 mg/kg, i.p.) has no effect on the sucrose-reinforced responding in the rat following 45 min of pretreatment compared to the vehicle controls. One-way ANOVA followed by Dunnet’s multiple comparisons test (F(2,18) = 1.113, *p* = 0.35). Data presented as mean ± SEM; n = 7 for each group.

Reduced responses to pleasurable stimuli such as sucrose is a preclinical measure of anhedonia. The traditional KOPr agonist U50488 has been shown to reduce the sucrose intake and seeking at 5 and 10 mg/kg [67]. We assessed the effect of Mesyl Sal B on sucrose-reinforced responding. Figure 5 shows that Mesyl Sal B at 0.3 and 1.0 mg/kg had no effect on the sucrose intake in male Sprague Dawley rats (F(2,18) = 1.113, *p* = 0.3501).

#### 2.5.2. Novel Object Recognition

**Figure 6:**
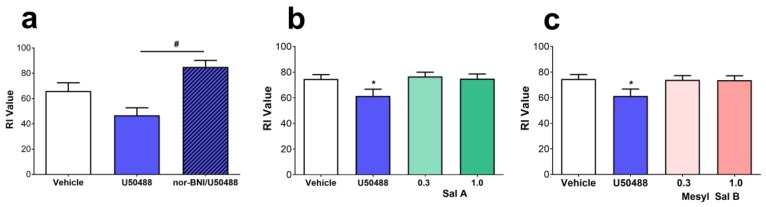
The effects of KOPr agonists on the novel object recognition task. (**a**) Rats treated with U50488 (10 mg/kg) decreased the recognition index (RI) value in the novel object recognition task which was prevented by the prior administration of nor-BNI (10 mg/kg, s.c.; n = 6). No change in the RI value was seen with either (**b**) Sal A or (**c**) Mesyl Sal B at either 0.3 or 1 mg/kg. One-way repeated measures ANOVA with Dunnett’s post hoc tests. Data presented as mean ± SEM; n = 22 for each KOPr treatment groups (* *p* < 0.05 compared to vehicle; # *p* < 0.05 compared to U50488).

Recognition memory has been shown to be impaired by stress and is also induced via KOPr dependent mechanisms [68] and following administration of U50488. Figure 6a shows a decrease in recognition index times following U50488 (10 mg/kg/i.p.), that was prevented by the prior administration of the KOPr agonist nor-BNI (F(1.62,9.311) = 8.394, *p* = 0.0089). In contrast, neither Sal A (Figure 6b), nor Mesyl Sal B (0.3 or 1 mg/kg) (Figure 6c) produced any impairment in memory in the novel object recognition task.

#### 2.5.3. Motor Coordination

**Figure 7:**
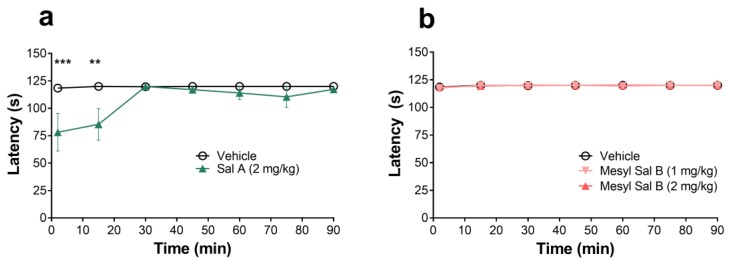
The rotarod motor coordination test in mice (**a**) Sal A shows a dose effect on motor impairment in the mouse with a decreased latency to fall (F(6,72) = 2.774, *p* = 0.0175). Post hoc analysis revealed 2 mg/kg Sal A causes significant motor impairment at 2 and 15 min. (**b**) Mesyl Sal B has no effects on the latency to fall in the rotarod test (F(12,96) = 0.1391, *p* = 0.9997). Two-way repeated measures ANOVA followed by the Bonferroni post-tests. Data presented as mean ± SEM; n = 6–7 for each group (** *p* < 0.01; *** *p* < 0.001 for 2mg/kg dose compared to vehicle).

The rotarod performance test was used to assess the motor coordination of mice following an i.p. injection of the vehicle, Sal A, or Mesyl Sal B. Two-way repeated measures ANOVA showed no significant interaction of treatment x time for the Mesyl Sal B experiment (F(12,96) = 0.1391 *p* = 0.9997). However, following Sal A administration, there was a significant treatment x time interaction (F(6,72) = 2.774, *p* = 0.0175). Sal A at a dose of 2 mg/kg showed a short-acting but significant motor impairment in the rotarod test in mice at 2 (*p* = 0.0005) and 15 min (*p* = 0.0039), whereas, Mesyl Sal B showed no motor impairment over a 90 min time period at either 1 or 2 mg/kg doses (Figure 7).

#### 2.5.4. Aversion

**Figure 8:**
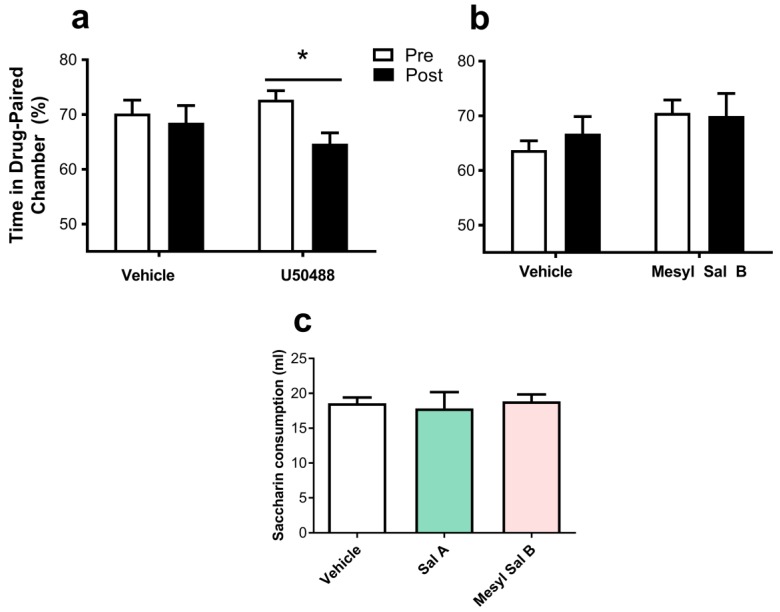
The effects of Mesyl Sal B on taste aversion and place aversion in rats. (**a**) Place aversion tests show that U50488 (10 mg/kg) significantly decreases the time spent in the drug-paired chamber, whereas (**b**) Mesyl Sal B (0.3 mg/kg) had no effects on the time spent in the drug-paired chamber. Paired *t*-tests (n = 7–10). (**c**) Evaluation of taste aversion shows no effect on the saccharin consumption on test day following Sal A (0.3 mg/kg), Mesyl Sal B (0.3 mg/kg) or the vehicle (F(2,16) = 0.1154, *p* = 0.8918) (n = 6–7). Data presented as mean ± SEM (* *p* < 0.05).

To determine the effects of Mesyl Sal B on aversion, we used two behavioral models, CTA and CPA. In tests measuring conditioned place aversion in rats, U50488 caused significant aversion to the paired chamber (*p* < 0.05), whereas Mesyl Sal B (0.3 mg/kg) did not significantly alter the amount of time spent in the drug-paired chamber (*p* = 0.767) (Figure 8a). Additional evaluation of the aversive effects of KOPr agonists utilized CTA tests in rats to compare the aversive effects of Mesyl Sal B with Sal A. Figure 8c shows the amount of novel tasting saccharin solution (0.1%) consumed by rats on test day with either Sal A or Mesyl Sal B pre-treatment and compared to vehicle-treated controls (F(2,16) = 0.1154, *p* = 0.8918). Acute exposure to Mesyl Sal B (0.3 mg/kg) did not induce taste aversion in rats further supporting the absence of aversive effects with Mesyl Sal B.

#### 2.5.5. Depression and Anxiety

**Figure 9:**
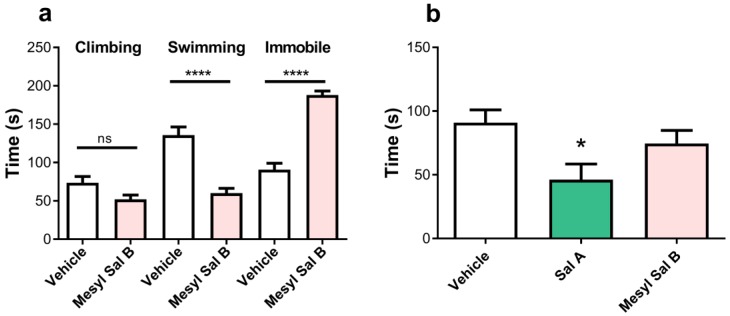
The effects of Mesyl Sal B in the forced swim test and elevated plus maze. (**a**) Mesyl Sal B attenuates the swimming time and increased immobility in the forced swim test (FST) in rats (n = 5–7). (**b**) There was no effect on the time spent in the open arm of the elevated plus maze following the administration of Mesyl Sal B (0.3 mg/kg), however, in contrast, Sal A (1 mg/kg) significantly decreased in the open arm time (n = 15–29). Data presented as mean ± SEM (* *p* < 0.05; **** *p* < 0.0001; ns = not significant).

The FST measures despair-like behaviors. Figure 9a shows the effect of the acute exposure of Mesyl Sal B (0.3 mg/kg) on forced swimming behaviors in rats. Mesyl Sal B pre-treated rats showed a significant decrease in the swimming time (*p* = 0.006) and a significant increase in the time spent immobile (*p* = 0.006) compared to vehicle pre-treated controls. This indicated that pro-depressive effects are produced by Mesyl Sal B in rats at doses that attenuated the effects of cocaine.

Time spent in the open-arm during a 5 min test in EPM tests is a preclinical model of anxiety [69], with decreases in open-arm times consistent with anxiogenic effects. Figure 9b reveals that Mesyl Sal B (0.3 mg/kg) has no effect on the time spent in the open arm (*p* > 0.9999) whereas Sal A (1 mg/kg), significantly decreased the time spent in the open arm (*p* = 0.0435).

## 3. Discussion

### 3.1. The Cellular Signaling Profile of Mesyl Sal B

Sal A was the first naturally-occurring non-nitrogenous KOPr agonist to be discovered [28,29]. The compound was isolated as the active ingredient of *Salvia divinorum*, a psychoactive plant native to Mexico [70,71]. Sal A has been shown to have anti-rewarding and antinociceptive effects in preclinical models [33,72,73,74,75,76,77], although Sal A is unsuitable for further development in the clinic due to its hallucinogenic and aversive properties [30,63,64,78], as well as a short duration of action [35,36,37]. However, analogues of Sal A have been developed, with the intention of improving the pharmacokinetic profile and reducing the side-effects [48,79,80,81]. We have previously shown that Mesyl Sal B has a longer duration of action using the warm-water tail withdrawal assay in mice [24], however, the underlying cellular signaling profile had not been previously assessed. Recently, there has been much interest in the concept of biased signaling [22,82,83], whereby the KOPr can preferentially activate desired G-protein signaling pathways associated with the therapeutic effects, rather than the β-arrestin2 pathways associated with some of the negative side-effects. In the present study, we have shown that Mesyl Sal B has a G-protein biased signaling profile (Table 1). Interestingly, in these cellular assays, Sal A also displays G-protein bias, which is in contrast to previous studies [22,84], although we have used different cell lines and functional assays to evaluate the signaling pathways. For instance, Sal A has been shown to be a balanced agonist in human embryonic kidney cells [22], whereas the present study utilized the U2OS and CHO cell lines. In addition, the importance of understanding the biased signaling in different species has recently been highlighted. DiMattio et al. [84] found that Sal A was significantly biased towards the β-arrestin2 pathway at the human receptor but had a similar activation of both pathways at the mouse KOPr. Furthermore, the KOPr agonist nalfurafine showed extreme G-protein biased signaling at the human KOPr as opposed to bias to a much lesser extent at the rat KOPr [83]. Overall, the finding that Mesyl Sal B has a G-protein bias is promising, however, further studies into the complex signaling pathways and the effects in different cell lines and species are warranted. 

### 3.2. Anti-Cocaine Effects of Mesyl Sal B

Despite immense efforts, researchers have not yet developed a successful FDA approved pharmacotherapy for psychostimulant abuse. KOPr agonists have been identified as potential therapeutic targets as the activation of the KOPr produces anti-rewarding effects [3,4] by decreasing dopamine levels in the nucleus accumbens [11,12,13]. We have previously shown that Sal A and Mesyl Sal B attenuate cocaine-prime induced reinstatement of drug seeking in rats [24,33]. Therefore, the current study aimed to expand the anti-cocaine profile of Mesyl Sal B to assess the effect on cocaine-induced hyperactivity and behavioral sensitization to cocaine. Previously, U50488 and U69593 have been shown to attenuate cocaine-induced hyperactivity and behavioral sensitization in rats [5,8], whereas Sal A suppressed cocaine sensitization but increased the locomotor activity produced by acute cocaine administration [72]. When administered for 45 min, but not 5 or 30 min, prior to cocaine, Mesyl Sal B (0.3 mg/kg) suppressed cocaine-induced hyperactivity (Figure 2). The results show that Mesyl Sal B has a prolonged onset of action in rats and is consistent with other studies demonstrating long-lasting effects in attenuating alcohol drinking behavior over 4 h [50] and a longer duration of action in antinociceptive assays compared to Sal A [24]. Similarly, Mesyl Sal B (0.3 mg/kg) attenuated behavioral sensitization to cocaine as shown by a reduction in the total ambulatory counts (Figure 2), consistent with previous work that showed Sal A (0.3 mg/kg) attenuated cocaine-induced behavioral sensitization in rats [72].

### 3.3. Antinociceptive Effects of Mesyl Sal B

The *Salvia divinorum* plant has traditionally been used in the spiritual practice of the Maztec Indians to produce hallucinogenic experiences, as well as in treating a variety of ailments including headaches, diarrhea, and rheumatism [70,71]. Sal A has been extensively tested in models of pain, showing particular efficacy in inflammatory models of nociception [73,74,77,85]. Therefore, the current study aimed to assess the antinociceptive effects of the Sal A analogue, Mesyl Sal B. We have previously published the warm-water tail withdrawal assay results for Mesyl Sal B showing a nociceptive duration of action of 60 min, as opposed to the 15 min duration of Sal A [24], indicating that Mesyl Sal B has an improved metabolic profile and bioactivity in vivo compared to Sal A. The present study used the same assay to assess the cumulative dose-response effects of the KOPr agonists. The results show that Mesyl Sal B exerts only partial effects compared to Sal A and U50488 (Figure 3), which is in contrast to the results from in vitro cellular assays showing that U50488, Sal A, and Mesyl Sal B are all full agonists at the KOPr [41,48]. In comparison, another C-2 analogue of Sal A, β-tetrahydropyran Sal B, was shown to be more potent and more efficacious than Sal A using the same dose-response procedure [77]. Similarly, the structurally-rigid spirobutyrolactone Sal B had a similar potency and efficacy in the cumulative warm-water tail withdrawal assay [81]. The reduced in vivo efficacy of Mesyl Sal B in assays of spinal-mediated antinociception contrasts to its potent anti-cocaine effects. Both behaviors require central nervous system active drugs, therefore, the differences are not due to the ability to cross the blood-brain barrier and may reflect site-specific differences in KOPr-mediated signaling pathways mediating particular behavioral effects. Interestingly, both the anti-addiction effects [86,87] and the antinociceptive effects of KOPr agonists [22,23] are believed to be due to G-protein signaling as the KOPr-mediated modulation of the DAT function is pertussis toxin-sensitive and dependent on the phosphorylation of extracellular signal-regulated kinase 1/2 (ERK1/2) [14].

To further assess the antinociceptive effects of Mesyl Sal B, the intraplantar 2% formaldehyde model of inflammatory pain was utilized. Sal A produced a significant reduction in the level of nociceptive and inflammatory pain, whilst Mesyl Sal A was effective at fewer time points (Figure 4). In addition, Sal A reduced the formaldehyde-induced paw edema, whereas Mesyl Sal B did not (Figure 4). This data contrasts with previous studies on C-2 modified Sal A analogues, including β-tetrahydropyran Sal B, that produced potent antinociceptive effects in the intraplantar 2% formaldehyde model [77]. The spirobutyrolactone Sal B analogue had potent antinociceptive effects in the inflammatory phase of the intraplantar 2% formaldehyde assay and significantly reduced formaldehyde-induced paw edema, however, did not reduce the nociceptive phase of pain [81]. The previously published warm-water tail withdrawal data does show that Mesyl Sal B has a longer onset of action, with effects only seen from 30–60 min [24]. Since the present study only had a pretreatment time of 5 min, a greater antinociceptive effect may have been produced with a longer pretreatment period, however, this does not fully explain the lack of efficacy in the inflammatory phase of pain.

The combined results from the spinally-mediated thermal model and the nociceptive and inflammatory phases of pain in the intraplantar formaldehyde model confirms the limited antinociceptive effects of Mesyl Sal B. These findings may indicate that the substitution of a mesylate group at the C-2 position results in differences in the downstream signaling pathways, however, further investigations into the in vitro pharmacology of Mesyl Sal B are warranted.

### 3.4. Side-Effect Profile of Mesyl Sal B

To develop clinically viable anti-cocaine or antinociceptive therapeutics, novel KOPr agonists need to have reduced side-effects compared to the traditional agonists U50488 and U69593, as well as the parent compound Sal A. Anhedonia is a side-effect associated with KOPr activation, which has been attributed to decreased levels of dopamine within the nucleus accumbens [88]. In preclinical models, anhedonia can be measured by a reduction in responses to a pleasurable stimulus such as sucrose. The traditional KOPr agonists, U50488 and spiradoline, suppress water consumption at the same doses that attenuate cocaine self-administration in rats (5, 10 mg/kg) [4]. In addition, intravenous infusions of bremazocine (0.0032 mg/kg/h), enadoline (0.001, 0.0032 mg/kg/h), ethylketazocine (0.01, 0.0032 mg/kg/h), and U50488 (0.1 mg/kg/h) not only decreased cocaine self-administration but also food reinforcements in rhesus monkeys [19,89,90]. In the present study, we have shown that Mesyl Sal B (0.3 and 1.0 mg/kg) had no effect on sucrose intake (Figure 5). This finding is supported by another study showing Mesyl Sal B at a higher dose of 3 mg/kg had no effect on sucrose or saccharin intake in mice [50]. Previous studies performed using Sal A also show no effects on sucrose self-administration at 0.3 mg/kg [72]. The lack of anhedonia effects exhibited by Mesyl Sal B contrast to the effects seen with the more potent Sal A analogue 2-methoxy methyl Sal A (MOM Sal B) [43,91]. MOM Sal B attenuates sucrose reinforcement in rats at a dose of 0.3 mg/kg [47]. However, since we also evaluated Mesyl Sal B at a higher dose of 1 mg/kg, these effects cannot be solely attributed to the increased potency of MOM Sal B. Other studies have also shown mixed effects of KOPr agonists on sucrose intake, with U50488 (administered intracerebroventricular) showing increased sucrose intake in rats [92,93], highlighting potential differences between different KOPr agonists in modulating natural reward behaviors. However, we have shown that Mesyl Sal B attenuates cocaine-induced behaviors without inducing anhedonia-like adverse effects in laboratory animals.

To further assess the side-effects of Mesyl Sal B, a novel object task was utilized as a measure of recognition memory. Animal studies have found that the endogenous KOPr agonist dynorphin causes spatial memory impairment [94] and KOPr activation is responsible for stress-induced learning and memory impairments [68]. We have shown that U50488 (10 mg/kg) leads to a decrease in the recognition index in the novel object recognition task (Figure 6). In contrast, neither Sal A nor Mesyl Sal B (0.3 or 1.0 mg/kg) produced any learning and memory impairments (Figure 6). These results are consistent with previous studies showing U50488 induces memory impairment in the passive avoidance test in mice [95]. Furthermore, disruption of the novel object recognition task has been shown previously for U50488 administered at 0.3 and 5.0 mg/kg in mice [96,97], suggesting that the disruption of cognitive function occurs at a wide range of doses, including at doses that do not impair motor coordination. Part of the pathway involved in long-term memory formation and retrieval is cAMP response element binding protein (CREB), however, different isoforms of CREB can either enhance or impair memory [98]. Following KOPr activation, CREB is induced as part of the β-arrestin2 pathway [99], whereas the G-protein pathway leads to a reduction in cAMP [100] and, therefore, inhibits CREB activation. This suggests that the balanced KOPr agonist U50488 may activate the isoform of CREB associated with the repression of memory via the β-arrestin2 signaling cascade. We have shown that Sal A and Mesyl Sal B are G-protein biased (Table 1), perhaps explaining the lack of memory impairment.

Another side-effect associated with the activation of the KOPr is motor incoordination or sedation [64]. Using the inverted screen test to evaluate motor coordination in mice, Sal A (0.5–2 mg/kg) and U69593 (1 mg/kg) have been shown to rapidly disrupt climbing behavior, however, the effects were short-lived [64]. Similarly, Sal A (3–10 mg/kg) induced motor incoordination in the rotarod performance test for 30 min, compared to the G-protein biased agonist RB-64 that did not cause any motor deficits [22]. In Rhesus macaques, Sal A (0.01–0.1 mg/kg i.v.) had a sedative effect, as measured by an unresponsiveness to environmental stimuli and postural relaxation [36]. In the current study, the rotarod performance test was also used to assess motor coordination in mice. Sal A (2 mg/kg) showed a short-acting but significant motor impairment in the rotarod test at 2–15 min, whereas, Mesyl Sal B (1 and 2 mg/kg) showed no motor impairment over a 90 min time period (Figure 7). This data is consistent with the effects of Mesyl Sal B (0.3 mg/kg) on spontaneous open field activity in drug naïve rats, where there was no significant effect of Mesyl Sal B on locomotor activity [24]. However, in the same open field test, we previously showed that Sal A (0.3 mg/kg) did not alter spontaneous locomotion in rats [72], indicating that the differences in sedative effects only occur at higher doses. These results show that Mesyl Sal B has no motor coordination impairments in mice when compared to the same dosage of the parent compound Sal A.

Previous studies have used CPA to measure the aversive properties of the traditional KOPr agonists U69593 [101,102,103] and U50488 [104]. Sal A has previously been shown to have no aversion in CTA experiments at 0.3 mg/kg [72], and conditioned place preference at low doses (0.1–40 μg/kg) but aversion at higher doses in rats (0.16–1 mg/kg) [105,106] and in mice (1–3.2 mg/kg) [63]. While we confirm the aversive properties of U50488 (10 mg/kg) (Figure 8a) seen in previous studies [104], Mesyl Sal B (0.3 mg/kg) had no effects on either place aversion (Figure 8b) or taste aversion (Figure 8c). Similar results were found for another more potent Sal A analogue, 2-ethoxymethyl ether Sal B (0.1 mg/kg), which did not show aversive effects in the CPA paradigm [34]. This further supports the proposition that it is possible to modulate drug-seeking at doses that do not have aversive effects, with both Sal A and Mesyl Sal B showing no significant aversive effects. Furthermore, the anxiogenic effect of Mesyl Sal B was measured using the EPM model. Interestingly, Sal A (0.16 mg/kg) has previously been shown to have anxiolytic effects in rats [107]. However, Mesyl Sal B (0.3 mg/kg), in contrast to Sal A (1 mg/kg), did not reduce the time spent on the open arm (Figure 9). The lack of effects on Mesyl Sal B in EPM is further evidence that unique structural Sal A analogues, such as Mesyl Sal B, have differences in preclinical behavior models that cannot be easily correlated to potency and efficacy in vitro.

Finally, the despair-like behaviors were measured in the FST model of pro-depressive effects. Mesyl Sal B reduced the swimming time and increased immobile behaviors, indicating pro-depressive effects at the same dose that attenuated cocaine behaviors (Figure 9). Previous findings show that Sal A (0.25–2 mg/kg) induces depressive effects without suppressing locomotion activity in rats [30,72]. Similarly, another Sal A analogue, MOM Sal B (0.3 mg/kg) showed pro-depressive effects [47], whereas 2-ethoxymethyl ether Sal B (0.3 mg/kg) and β-tetrahydropyran Sal B (2 mg/kg) did not [34]. Since a reduction in swimming time is related to the modulation in serotonin transporter function [108], depressive effects produced by these novel neoclerodanes may also involve alterations in central serotonin systems. However, the results are complicated and the pro-depressive effects should be interpreted with caution, as Sal A (10–1000 μg/kg) has been shown to have both potent anti-depressive and anxiolytic effects in rats in previous studies [107,109].

### 3.5. Limitations and Future Directions

KOPr agonists have great potential as anti-addictive or analgesic therapeutics [22,24,33,47,72,77], however, the aversive side-effects have prevented their clinical development. Several strategies have been employed to reduce the side-effect profile of KOPr agonists, including the development of G-protein biased agonists [22,23,24,25] and peripherally restricted KOPr agonists [26,27]. Nalfurafine, a potent and selective KOPr agonist is the first KOPr agonist to be approved for use in humans, currently being prescribed to hemodialysis patients for the treatment of medication-resistant pruritus [110,111]. Importantly, nalfurafine was recently found to be extremely G-protein biased at the human KOPr [83], and has little abuse potential [112,113]. In the present study, we identified that Mesyl Sal B was G-protein biased (Table 1), perhaps explaining the reduced side-effect profile. However, we only assessed one G-protein signaling pathway (inhibition of forskolin-induced cAMP accumulation) and one β-arrestin pathway (β-arrestin2 recruitment). To gain a further understanding of the signaling pathways involved and the bias factor, more downstream proteins could be evaluated to create a ‘web of bias’ for Mesyl Sal B.

## 4. Materials and Methods

### 4.1. Compounds and Drugs

Cocaine HCl (BDG Synthesis, Wellington, New Zealand) was dissolved in 0.9% saline. Sal A was isolated from dried Salvia divinorum leaves [114] and Mesyl Sal B synthesized from Sal A (>98% pure by HPLC) [41]. Nor-binaltorphimine (nor-BNI) and (trans-(±)-3,4-dichloro-*N*-methyl-N(2-[1-pyrrolidinyl]-cyclohexyl) benzenacetamide methanesulfonate (U50488) were obtained from Sigma-Aldrich (Auckland, New Zealand). The doses of the KOPr agonists [24,33,72] and antagonists [115] were based on previous studies. Drugs were suspended in either 75% dimethyl sulfoxide (DMSO) for the cocaine hyperactivity, behavioral sensitization, sucrose self-administration, CTA and FST procedures; a 2:1:7 ratio of DMSO: Tween-80: water for the novel object recognition, CPA and EPM procedures; a 2:1:7 ratio of DMSO: Tween-80: saline (0.9%) for the warm-water tail withdrawal; or a 1:4:5 ratio of DMSO: propylene glycol: phosphate buffered saline (PBS) for the intraplantar formaldehyde assay and rotarod procedures. All solutions were administered via intraperitoneal (i.p.) or subcutaneous (s.c.) injection, with the final volume made up to 1 mL/kg in rats and 10 mL/kg in mice. All drug weights refer to the salt.

### 4.2. Cellular Assays

The PathHunter™ U2OS cell line was purchased from the DiscoveRx Corporation (Fremont, CA, USA) and stably expresses β-arrestin2 and hKOR (U2OS-hKOR-βarrestin2-DX). These cells were cultured in a Minimum Essential Medium (MEM) with 10% fetal calf serum, 1% penicillin/streptomycin, 500 µg/mL G418 and 250 µg/mL hygromycin B and grown at 5% CO_2_, 95% relative humidity and 37 °C. The PathHunter™ assay was performed according to the manufacturer’s protocol. Briefly, cells were plated overnight using an AssayComplete™ Cell Plating 5 Reagent (DiscoveRx, Fremont, CA, USA) in 384 well clear bottom plates (Grenier Bio One, North Carolina, NC, USA; Item No. 788093) at a density of 20,000 cells/well. Cells were treated with KOPr agonist doses for 30 min at 37 °C and then a fresh plating reagent and PathHunter™ detection reagent (DiscoveRx Corporation, Fremont, CA, USA) was added and incubated for 60 min at room temperature. Chemiluminescence was detected using an EnSpire Multimode Plate Reader-2300 (PerkinElmer, Waltham, MA, USA).

Assays evaluating attenuation of cAMP levels utilized the HitHunter™ CHO cell line (DiscoveRx Corporation, Fremont, CA, USA) and were performed as described previously [81]. Data were analyzed using a nonlinear regression analysis in GraphPad Prism 7 (GraphPad Software, La Jolla, CA, USA). cAMP accumulation data were normalized to the vehicle and forskolin-only control values. β-arrestin-2 recruitment data were normalized to the vehicle and U50488 maximum response values. All compounds were run in parallel assays in triplicate or quadruplicate in ≥2 individual experiments. As previously published [79], calculations of bias were determined using the following equation, where U50488 was the control compound.

(1) $$\;\log\left(\mathrm{bias}\;\mathrm{factor}\right)=\log{\left(\frac{E_{\max\left(\mathrm{test}\right)}\times {\;\mathrm{EC}}_{50\left(\mathrm{control}\right)}}{{\mathrm{EC}}_{50\left(\mathrm{test}\right)}\times {\;E}_{\max\left(\mathrm{control}\right)}}\right)}_{\beta -\mathrm{arrestin}}-\log{\left(\frac{E_{\max\left(\mathrm{test}\right)}\times {\;\mathrm{EC}}_{50\left(\mathrm{control}\right)}}{{\mathrm{EC}}_{50\left(\mathrm{test}\right)}\times {\;E}_{\max\left(\mathrm{control}\right)}}\right)}_{G-\mathrm{protein}\;}\;$$

Using this formula, a bias factor of 1 is a balanced agonist, less than 1 is a G-protein biased agonist and more than 1 is a β-arrestin2 biased agonist.

### 4.3. Animals

Male Sprague-Dawley rats (200–300 g) and adult male B6.SJL protein tyrosine phosphatase receptor type c allele a (ptprca) mice (23–30 g) were housed within the Victoria University of Wellington animal facilities. All animals were group housed in polycarbonate cages in temperature (19–21 °C) and humidity (55% relative humidity) controlled rooms. Lights were maintained on a 12:12 h cycle with lights on at 07:00 h. Unless stated otherwise, all animals were given free access to food and water except during experimental sessions. All animals were handled for at least 3 days prior to experimentation to acclimatize the animals to handling and prevent stress during experimental procedures. The laboratory care of animals and experimental procedures were reviewed and approved by the Victoria University of Wellington Animal Ethics Committee and were carried out in agreement with the New Zealand Animal Welfare Act, 1999.

### 4.4. Cocaine-Induced Hyperactivity and Behavioral Sensitization

The dose of Mesyl Sal B (0.3 mg/kg) was chosen based on the lowest effective dose that has previously been shown to attenuate drug seeking/taking behavior in the rat [24]. For cocaine-induced activity tests, drug naïve rats were initially injected with either vehicle (75% DMSO, 1 mL/kg, i.p.) or Mesyl Sal B (0.3 mg/kg, i.p.) followed 45 min later with either 0.9% saline (1 mL/kg, i.p.) or cocaine (20 mg/kg, i.p.) and locomotion activity determined in an open field chamber (Med Associates ENV-520; St. Albans, VT, USA) in 5 min intervals for 60 min. The expression of locomotor sensitization experiments was performed as previously described for Sal A [72]. Briefly, drug naïve rats were treated with either 0.9% saline (1 mL/kg, i.p.) or cocaine (20 mg/kg, i.p.) once daily for 5 consecutive days and were immediately returned to their home cage. On days 6–9, the animals were drug-free and remained in their home cage. On day 10, the effect of Mesyl Sal B pre-treatment was tested on the expression of cocaine sensitization. Rats were injected with either a vehicle (75% DMSO, 1 mL/kg, i.p.) or Mesyl Sal B (0.3 mg/kg, i.p.) followed, 45 min later, by cocaine (20 mg/kg, i.p.) and the locomotion activity was measured for 60 min. The dose of cocaine was selected based on previous reports, which showed that cocaine administration (20 mg/kg, i.p.), once daily for 5 consecutive days, produced locomotor sensitization in rats [8,53].

### 4.5. Warm-Water Tail Withdrawal

The cumulative dose-response effects of the KOPr agonists were measured using the warm-water tail withdrawal assay as previously described [77,81]. Mice were habituated to the Plexiglas restrainers (internal diameter 24 mm) for 15 min daily for the 4 days prior to the experiment. Withdrawal latencies were measured by immersing one-third of the tail in a warm water bath (50 ± 0.5 °C) and the time to withdraw recorded. To avoid tissue damage, a maximum cut-off latency of 10 s was used. Three measurements were averaged for the baseline latency of each individual mouse. The maximum possible effect (% MPE) was calculated using the following formula:

(2) $$\;\%\;\mathrm{MPE}=\;\left(\frac{\mathrm{test}\;\mathrm{latency}-\mathrm{baseline}\;\mathrm{latency}}{10-\mathrm{baseline}\;\mathrm{latency}}\right)\;\times \;100\;$$

The test latencies were measured at 30 min following each subcutaneous (s.c.) injection of KOPr agonist (delivered at 5 μL/g of weight), and the next escalating dose administered, therefore evaluating the dose-response effects using a within animal design. For Sal A and Mesyl Sal B, the following cumulative doses were assessed: 0.3, 0.6, 1.0, 2.5, 5.0, 7.5, and 10 mg/kg. A non-linear regression analysis was used to determine the potency (EC_50_) and efficacy (E_max_) of the data compared to the known full KOPr agonist U50488.

### 4.6. Intraplantar Formaldehyde Inflammatory Pain Model

The procedure was carried out as previously described [77,81]. The apparatus consisted of a box (27.5 × 18.5 cm) placed on a glass surface with a 45° angled mirror placed underneath to facilitate observations of the hind paws via a digital video camera. The mice were habituated to the test enclosure for 15 min before testing. The mice were given the KOPr agonist or vehicle treatment via i.p. injection 5 min prior to the administration of 20 μL of formaldehyde (2% in PBS) or PBS control via intraplantar injection. The pain behaviors were recorded for 60 min. The methods of Dubuisson and Dennis [116] were used to assess the pain behavior of the injected paw using a weight-bearing score. The pain was scored as 0 if the mouse showed normal behavior; 1 for partial weight-bearing with only the digits touching the floor; 2 with no weight-bearing with the paw raised; and 3 where the paw was bitten, licked or shaken. Pain behavior scores were assigned at 5 s intervals for 60 min by an observer blinded to the treatment group. An average pain score was calculated for each 5 min time period. For the area under the curve analysis, the two phases of pain were separated out as follows: 0–15 min phase 1 nociceptive pain; and 20–60 min phase 2 inflammatory pain.

### 4.7. Rotarod Motor Coordination Tests

Mice were initially trained to run on the rotarod (60 mm wide and 30 mm in diameter; Panlab, Harvard apparatus, Barcelona, Spain) at 16 rpm for 120 s. Mice that were able to successfully complete two consecutive trials from a maximum of three trials without falling were chosen for the experimental procedure. Mice were injected with either vehicle, Sal A or Mesyl Sal B and immediately placed on the rotarod at 16 rpm for 120 s. The time at which the mouse fell from the rotarod apparatus was recorded and the test repeated at 15, 30, 45, 60, 75, and 90 min using a within animal design.

### 4.8. Sucrose Self-Administration

For sucrose reinforcement tests, rats were maintained at approximately 85% of their initial feeding weights with free access to water throughout the experiment. Rats were trained to orally self-administer a 10% sucrose solution in eight modular test chambers (Med Associates) as previously described [47]. Rats were trained to self-administer sucrose orally using an auto-shaping procedure (45 min daily for ten days). Once stable responding was achieved, the animals were maintained on a Fixed Ratio 1 (FR1) schedule of reinforcement where depression of the active lever (left lever) led to the delivery of sucrose solution. Following oral acquisition, the response requirements were increased to an FR5 schedule of reinforcement. Daily 1 h sessions were conducted until there was less than 20% variation in responding for three consecutive days. The animals on stable FR5 reinforcements were subjected to the sucrose reinforcement test. On the test day, rats received either vehicle (75% DMSO, 1 mL/kg, i.p.) or Mesyl Sal B (0.3, 1.0 mg/kg, i.p.) and 45 min later, sucrose reinforced lever-press responding was recorded for 60 min.

### 4.9. Novel Object Recognition

The effect of KOPr agonists on learning and memory were evaluated in the novel object recognition tasks based on the methods of Schindler et al. [97]. Briefly, male Sprague Dawley rats were handled on days 1–2 days prior to 3 days of habituation in the empty test box for 30 min daily (days 3–5). On day 6 rats were familiarized to the constant object by securing two identical objects on either side of the test box (3 times for 6 min each with an interval time of 10 min). Long-term memory was tested 24 h following familiarization (day 7) following the i.p. injection of either treatment or vehicle and time spent exploring the novel and familiar object recorded using Smart software (Panlab, Harvard Apparatus). The recognition index (RI) was calculated as N/(F + N)) × 100, where N is the time (in seconds) spent with the novel object and F is time spent with the familiar object. Rats that only interacted with a single object were excluded. A within animal subject design using a balanced Latin square design was performed with a one-week rest period between each drug administration (n = 24 with 2 exclusions (n = 22 per treatment)). Due to the long-acting effects of nor-BNI in vivo, a group of 6 rats were administered nor-BNI (+U50488) at the end of the experiment to evaluate whether the effects on novel object recognition were KOPr mediated.

### 4.10. Conditioned Taste Aversion

Rats had free access to food but were water deprived for 23 h (water habituation session) or 23 h 20 min (saccharin sessions) per day for the entire duration of the study. During habituation sessions, rats received water, whereas, on the pairing and test days, animals received a 0.1% novel tasting saccharin solution. On day 1, rats were placed on a water deprivation schedule for 23 h. The following day, animals were given access to water for 1 h. The amount of water consumed was measured daily and the process repeated until the variation in water consumption was ≤2 mL for three consecutive days. The next day, instead of water, the rats were presented to a novel tasting saccharin solution (0.1%). The amount of saccharin consumed was noted for 40 min. The animals were divided into two saccharin consumption matched groups and immediately treated with either vehicle (75% DMSO, 1 mL/kg, i.p.) or Mesyl Sal B (0.3 mg/kg, i.p.). The next day, the rats were given water for 60 min. On the following test day, the rats were presented with saccharin solution and consumption measured. In order to determine any taste aversion, the saccharin consumption in the Mesyl Sal B treated group on test day was compared with the vehicle-treated group.

### 4.11. Conditioned Place Aversion

CPA was measured using a three-chambered place preference chamber (LE890, PanLab; Harvard Apparatus) using previously described methods [103]. One large chamber was black and white striped with a black rough floor, while the other was white with black spots with a white smooth floor. Experiments were recorded via a video camera in the presence of white noise and the time spent in each chamber was tracked using the SMART software (version 3.0; PanLab; Barcelona, Spain). The rats were handled daily for several days prior to experimentation. On the first experimental day, the animals were placed in the corridor of the three-chamber box and allowed to freely move between chambers for 15 min, and the time spent in each chamber was recorded. Rats showing a greater than 80% preference for any chamber were excluded from the study. On day 2, the rats were injected with the vehicle or Mesyl Sal B (0.3 mg/kg, i.p.) with a volume of 1 mL/kg and immediately confined to their preferred chamber for 45 min. The following day (day 3), the animals were injected with the vehicle and confined to their non-preferred chamber for 45 min. Days 2 and 3 were repeated twice, a total of 3 times each. The time between the conditioning sessions ranged from 10 to 15 h. On day 8, the rats were placed in the corridor and allowed free access to both chambers for 15 min. The time in each box was measured and then the percent time in the drug-paired chamber was calculated.

### 4.12. Forced Swim Test

The FST apparatus consisted of a cylinder (44 cm high and 20 cm in diameter) filled to 35 cm with water maintained at 25 ± 1 °C. On the habituation day, the rats were placed in the swim chamber for 15 min and the following day were administered either with Mesyl Sal B (0.3 mg/kg, i.p.) or the vehicle (75% DMSO, 1 mL/kg, i.p.) and placed in the swim chamber45 min later. The forced swimming behavior was recorded by a video camera and the climbing, swimming, or immobility behaviors were recorded for 5 min. Data were analyzed using the SMART software (version 3.0; PanLab, Barcelona, Spain).

### 4.13. Elevated Plus Maze

The EPM was custom made according to the dimensions of Walf and Frye [117] with the addition of open arms having 2.5 cm high clear plastic sides and elevated to 55 cm. The rats were placed in the center of the apparatus facing an open arm and recorded by a video camera for 5 min. The time spent in the open arm was measured by an experimenter blinded to the treatment group.

### 4.14. Statistical Analysis

The GraphPad Prism software was used to determine statistical significance. Comparisons between treatments were analyzed using one-way analysis of variance (ANOVA). Data for sucrose intake, novel object recognition, CTA, FST, and total ambulatory counts for cocaine-induced hyperactivity and expression of sensitization experiments were performed using one-way ANOVA followed by the Dunnet’s or Bonferroni multiple comparisons post hoc tests. Comparisons of multiple effects were analyzed using two-way ANOVA. The time-course analysis was performed using repeated measures two-way ANOVA (treatment × time interaction) followed by a Bonferroni post hoc test (every 5 min interval) for cocaine-induced hyperactivity, expression of behavioral sensitization, and the intraplantar 2% formaldehyde tests and at times indicated for the rotarod performance test. Student *t*-tests were applied for CPA to compare pre- and post-test times, and in EPM to determine differences in time spent in open arm between control-treated rats. Data are presented as mean ± standard error of the mean (SEM) and were considered to reach statistical significance when *p* < 0.05.

## 5. Conclusions

The activation of KOPr decreases dopamine levels in the nucleus accumbens [11,12,13], producing anti-rewarding effects [3,4]. The psychoactive natural product Sal A is a potent and selective KOPr agonist isolated from the plant *Salvia divinorum* [28,29]. Sal A attenuates cocaine-prime induced drug seeking in rats [33], however, Sal A has a short duration of action [35,36,37] and is associated with negative side-effects [30,63,64,78,107], which has prevented its clinical development. The Sal A analogue, Mesyl Sal B, has a mesylate substitution at the C-2 position and has a similar binding affinity for the KOPr compared to Sal A and U69593 [41,45,49]. We have previously shown that Mesyl Sal B has a longer duration of action in the warm-water tail withdrawal assay in mice and Mesyl Sal B attenuates cocaine-prime drug seeking in rats [24]. Therefore, we aimed to further assess the anti-cocaine effects of Mesyl Sal B, as well as the side-effect profile and any potential antinociceptive effects.

We have determined that Mesyl Sal B has increased potency in vitro compared to U50488 in an assay measuring the inhibition of forskolin-stimulated cAMP accumulation, whereas, Mesyl Sal B has similar potency and efficacy to U50488 in an assay measuring β-arrestin2 recruitment. Together, this indicates that Mesyl Sal B has G-protein biased signaling in vitro. The behavioral effects of Mesyl Sal B in vivo are also unique. Mesyl Sal B was effective at attenuating cocaine-induced hyperactivity and behavioral sensitization to cocaine at similar doses to Sal A. In addition, Mesyl Sal B had fewer side-effects than Sal A and U50488 at the dose that modulated the cocaine behaviors. Mesyl Sal B produced no motor defects in the rotarod performance task; no memory impairment in the novel recognition task; no anxiety on the EPM; or aversion in the CTA or CPA tests. However, pro-depressive effects were observed in the FST. In contrast to Sal A, Mesyl Sal B was ineffective in behavioral models of antinociception. The cumulative dose-response effects in the warm-water tail withdrawal assay showed Mesyl Sal B only had a partial efficacy. This lack of antinociceptive efficacy was confirmed in the intraplantar formaldehyde model of inflammatory pain, where the effects of Mesyl Sal B were limited in comparison to Sal A. Overall, this study demonstrated that structurally novel Sal A analogues, such as Mesyl Sal B, have unique behavioral and cellular signaling properties, demonstrating that KOPr-mediated anti-rewarding effects, antinociceptive effects, and side-effects can be distinct. This holds promise for the development of KOPr therapeutics with fewer side-effects.

## Figures and Tables

**Table 1:** The activity of U50488, Sal A, and Mesyl Sal B in the cyclic adenosine monophosphate (cAMP) and β-arrestin recruitment assays.

Compound	Inhibition of Forskolin-Induced cAMP Accumulation (HitHunter^TM^)			β-Arrestin Recruitment (PathHunter^TM^)			
	Potency (nM)	Potency (SEM)	Efficacy (%)	Potency (nM)	Potency (SEM)	Efficacy (%)	Bias Factor
U50488	0.23	0.094	99.5	162.2	43.4	102.6	-
Sal A	0.03	0.013	98.7	248.5	66.5	94.9	0.35
Mesyl Sal B	0.12	0.049	101.2	236.0	44.3	90.2	0.61
